# Towards Heterogeneous Catalysis: A Review on Recent Advances of Depositing Nanocatalysts in Continuous–Flow Microreactors

**DOI:** 10.3390/molecules27228052

**Published:** 2022-11-20

**Authors:** Hao Feng, Ying Zhang, Jian Liu, Dong Liu

**Affiliations:** 1 MIIT Key Laboratory of Thermal Control of Electronic Equipment, School of Energy and Power Engineering, Nanjing University of Science & Technology, Nanjing 210094, China; 2 College of Petrochemical Engineering, Lanzhou University of Technology, Lanzhou 730050, China

**Keywords:** microreactor, catalyst synthesis, sol–gel method, bio–inspired electroless deposition, layer–by–layer self–assembly

## Abstract

As a promising technology, microreactors have been regarded as a potential candidate for heterogeneous catalytic reactions as they inherently allow the superior advantages of precise flow control, efficient reactant transfer, flexible operation, etc. However, the wide market penetration of microreactors is still facing severe challenges. One of the most important reasons is the preparation of a high–performance catalytic layer in the microreactor because it can directly influence the catalytic activity and stability the reactor and thus the deployment the microreactor technology. Hence, significant progress in depositing nanocatalysts in microreactors has been made in the past decades. Herein, the methods, principles, recent advances, and challenges in the preparation of the catalyst layer in microreactors were presented. A general description of the physicochemical processes of heterogeneous catalytic reactions in microreactors were first introduced. Then, recent advances in catalyst layer preparation in microreactors were systematically summarized. Particular attention was focused on the most common sol–gel method and its latest developments. Some new strategies proposed recently, including bio–inspired electroless deposition and layer–by–layer self–assembly, were also comprehensively discussed. The remaining challenges and future directions of preparing the catalytic layer in microreactors with high performance and low cost were highlighted.

## 1. Introduction

A microreactor refers to the microfluidic technology of allowing microchannels, microchambers, etc., with a feature size at the microscale (10 μm~1000 μm) to flexibly and precisely control reactants [1]. Due to their extremely large specific surface area, which can usually reach the magnitude of 10^5^ m^2^/m^3^ [2], microreactors can inherently play positive roles in facilitating the mass transfer of both gas and liquid reactants and products, thus dramatically enhancing the catalytic reaction and improving the conversion and selectivity [3,4,5]. Hence, in the past decades, microreactors have been introduced into various applications, including fine chemical synthesis [6,7], nanomaterials preparation [8], biochemical analyses [9,10], energy conversion and utilization [11,12], etc., and present excellent application potential in these fields.

**Figure 1:**
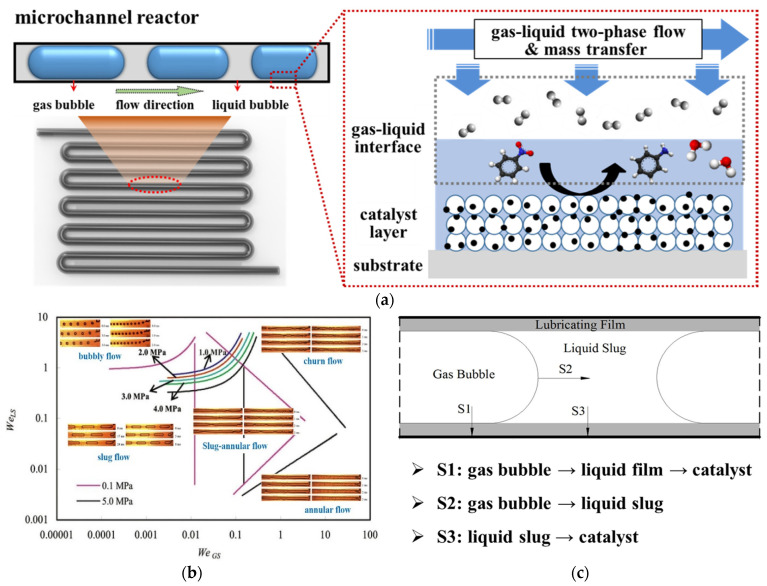
(**a**) Schematic description of physicochemical processes of heterogeneous catalytic reactions in microreactors. (**b**) Gas–liquid two–phase flow regimes in the microchannel, adapted from [15], Copyright 2013, with permission from Elsevier. (**c**) Various mass transfer sub–processes in a typical Taylor reacting flow in a microreactor.

Here, a common serpentine microchannel reactor was used to illustrate the detailed physicochemical processes of heterogeneous catalytic reactions in microreactors, as shown in Figure 1a. Typically, the cross–section of a microchannel can be circular, triangular, square, etc., and the catalyst is coated on the inner wall of the microchannel [13]. Once the gas and liquid reactants are introduced into the microchannel reactor, firstly, ascribing to the competition of interfacial tension, inertia forces, viscous forces, etc., for the gas and liquid phases, various gas–liquid two–phase flow regimes including bubbly flow, slug flow, annular flow, etc., form in the microchannel [14,15,16], see Figure 1b. Then, since the catalytic reaction is only allowed at the active sites of the wall–coated catalysts, the reactants have to transfer from the bulk to the catalytic surface through various interfaces, see Figure 1c. In this process, there exist various mass transfer sub–processes for both gas [17] and liquid [18] reactants. Subsequently, the reactants transferred from the bulk are first adsorbed at the active sites of the catalytic wall and are then proceeded to the catalytic reaction. Typically, due to the inherent complexity of heterogeneous catalytic reactions, there are many elementary reaction steps in this process. Thus, in addition to the desired product, various by–products or intermediates may also be generated at the catalytic wall. Finally, to guarantee the catalytic reaction, both the desired product and the by–products as well as the intermediates are desorbed from the active sites and then transferred from the catalytic wall to the bulk of the microreactor.

On the one hand, considering that the occurrence of heterogeneous catalytic reactions needs to overcome the activation energy barrier, the addition of a catalyst can promote the reaction by reducing the activation energy. The inherent performance of the catalyst layer then directly affects its catalytic activity and, consequently, the catalytic reaction rate [19]. Therefore, the preparation of catalyst layers in microreactors is particularly important. On the other hand, because the mass transfer characteristic, which is inherently associated with the gas–liquid two–phase flow in a microreactor, determines the supply of the reactant from the bulk to the active sites, it also then plays an important role in influencing the catalytic reaction [18,20]. As a consequence, the determinants for the performance of a microreactor can be attributed to the following two aspects: (1) the performance of the wall–coated catalyst in the microreactor and (2) the mass transfer characteristics of gas and liquid reactants in the microreactor. Both these two aspects can play significant roles in influencing the kinetic reaction rate, the reaction path, the stability, etc. [21,22,23].

To date, various review articles focusing on the fundamental multiphase flow behaviors and mass transfer characteristics in microreactors have been proposed. Kreutzer et al. [24] overviewed the fluid mechanics and mass transport phenomena of Taylor flow. Detailed analyses were discussed for both the mass transfer and pressure drop as well as the residence time distribution of Taylor flow in capillary microreactors. Haase et al. [25] presented an overall summarization of the hydrodynamics and mass transfer of Taylor flow regimes in microchannels. Various correlations to predict the hydrodynamics (e.g., bubble velocity, gas bubble and liquid slug lengths, gas holdup, and gas void fraction) and mass transfer coefficients between gas–liquid mass transfer and liquid–solid mass transfer as well as gas–solid mass transfer under both non–reaction and reaction conditions were systematically discussed. In addition, the advantages of microreactor technology in various areas including organic synthesis, biological analysis, nanoparticle preparation, energy conversion, etc., have also been comprehensively reviewed [8,11,26,27,28]. Regarding the preparation of the catalyst layer (i.e., the deposition of nanocatalysts) onto the inner wall of microreactors, although Meille [29] and Tanimu [30] summarized some traditional methods of depositing catalysts on a structured surface, it is still difficult to deposit efficient catalysts in the small spaces of microreactors. Considering that the rapid development of microreactor technology greatly broadens its application scope, various methods of preparing catalytic coatings in microreactors suitable for performing different heterogeneous reactions urgently need to be summarized. Hence, in this review, the objective was to provide a comprehensive summarization of the recent advances in catalyst layer preparation in microreactors. The most common sol–gel method and its development were first discussed. Then, the latest developed methods such as bio–inspired electroless deposition, layer–by–layer self–assembly, etc., were summarized. Finally, the remaining challenges and future directions of preparing catalytic coatings with a high performance and low cost in microreactors were also highlighted.

## 2. Catalysts Layer Preparation Based on the Sol–Gel Method

In multiphase catalytic reactions, the choice of catalyst depends on the requirements of the heterogeneous catalytic reactions. Common catalysts are metallic monomers such as nickel, copper, platinum, palladium, gold, silver, etc. [31]. Typically, the synthesis of a catalyst allowing both a high dispersion and high efficiency is of great importance to not only catalytic research but also industrial deployment [32]. To this end, the utilization of a catalyst support (e.g., Al_2_O_3_, SiO_2_, TiO_2_, zeolites, carbon materials) with a high specific surface area, thermal stability, and mechanical strength is also an attractive topic in heterogeneous catalysis [33]. Sol–gel is a method of preparing catalytic coatings in microreactors based on a chemical precursor solution (or colloidal dispersion). Since the aging process results in the gelation of the sol, which then presents a high viscosity, a thicker catalyst layer can then be prepared on the inner surface of the microreactor [34]. To meet the requirements of the different scenarios in microreactors, the sol–gel method for preparing the catalyst layer in a microreactor has also gradually developed into different forms.

A typical implementation process of the sol–gel method is to prepare a viscous chemical precursor that contains the catalytic species. Then, this highly viscous precursor is deposited in the microreactor with the help of calcination treatment [29]. For example, Liguras et al. [35] prepared a catalyst sol–gel by using Al[OCH(CH_3_)_2_]_3_, Ni(NO_3_)_2_⸱6H_2_O, and La(NO_3_)_3_⸱6H_2_O as precursors. By immersing monoliths or foams into the sol–gel, a thicker catalyst layer was obtained after calcination at 550 °C. Another type of sol–gel method is based on the dispersion of a finished material, i.e., the catalyst support or the catalyst itself [29]. In this method, the prepared catalyst is firstly dispersed in a solution to prepare the sol. Then, the sol is introduced into the microreactor to form the catalyst layer. So, this method is also known as the “suspension method”, but the difference with the traditional sol–gel method is tiny because this method also needs gelification steps in some cases [36].

**Figure 2:**
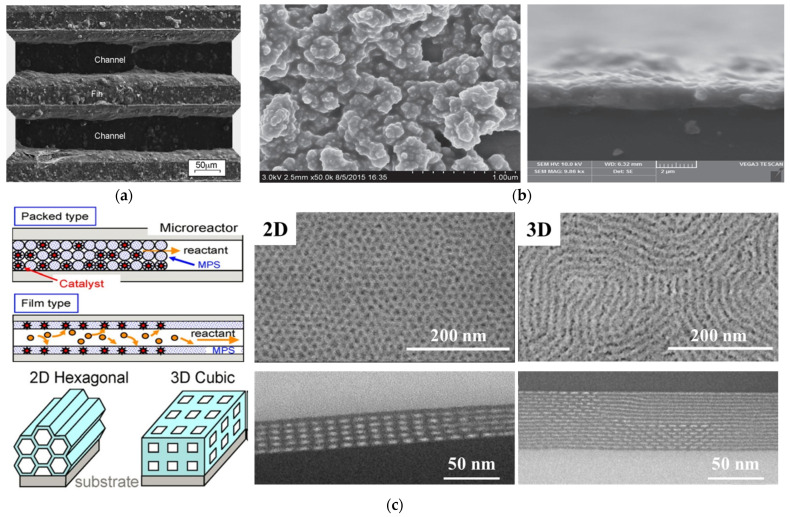
(**a**) Morphology of the catalyst layer in a reactor prepared by “suspension method”, adapted from [37], Copyright 2004, with permission from Elsevier. (**b**) Surface and cross–section images of Pd/γ-Al_2_O_3_ catalyst layer in a catalytic membrane microreactor, adapted from [38], Copyright 2016, with permission from Elsevier. (**c**) Schematic diagram of the formation of 2D and 3D catalyst layers and the corresponding high–resolution SEM and TEM images, adapted from [40], Copyright 2008, with permission from Elsevier.

As seen for example in the work of Pfeifer et al. [37], ZnO nanoparticles were used as the support for the impregnation of a Pd catalyst. The obtained Pd/ZnO nanocatalyst was then suspended with a magnetic stirrer for 24 h at 1100 rpm in an aqueous solution containing hydroxyl ethyl cellulose. After the formation of the suspension layer (i.e., the wet coating) in a microstructured reactor, the layer was dried and then calcined to obtain the sintered catalyst (see Figure 2a). Another work was proposed by Liu et al. [38], where highly dispersed colloidal Pd/γ-Al_2_O_3_ was employed as the catalyst and equipped in a catalytic membrane microreactor. In this case, the chemical reduction method was utilized to prepare the Pd nanocatalyst that was dispersed in an aqueous solution [39]. Then, the traditional impregnation process was used to prepare the Pd/γ-Al_2_O_3_ nanocatalyst by adding commercial γ-Al_2_O_3_ nanoparticles in the Pd nanocatalyst suspension. After centrifuging and drying, the obtained Pd/γ-Al_2_O_3_ catalyst was suspended in a polyvinyl alcohol (PVA) solution to prepare the catalyst sol. The sol was injected into the microreactor to form the catalyst layer with the help of a nitrogen purge and a subsequent drying process (see Figure 2b). Generally, a prepared catalyst layer using the above–mentioned sol–gel methods can present good catalytic activity for heterogeneous catalytic reactions. However, according to the preparation process, since part of the catalyst is embedded, the utilization efficiency of the catalyst may be restricted. Meanwhile, the mass transfer of both gas and liquid reactants to the active sites at the bottom of the catalyst layer will also be inhibited.

**Figure 3:**
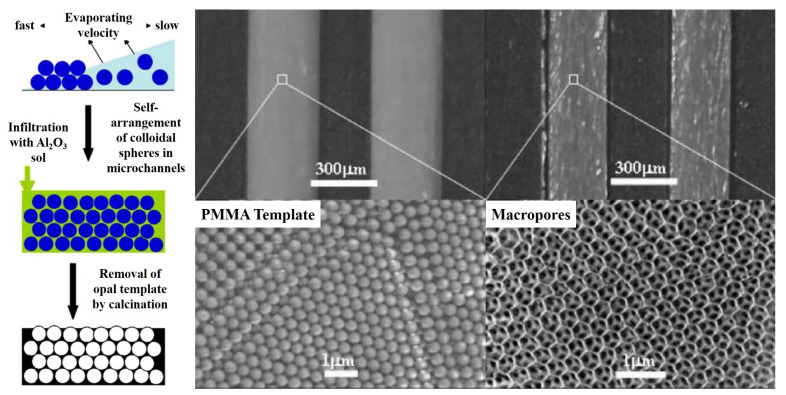
Preparation procedure and SEM images of the highly ordered macroporous structure of γ-Al_2_O_3_ support layer, adapted from [47,48], Copyright 2007 and 2008, with permission from Elsevier and RSC.

To solve this issue, the impregnation–reduction process was introduced into the sol–gel method [41]. Different from the traditional sol–gel method, the procedure of this modified method can be divided into the following three steps: (1) the preparation of a support layer in the microreactor, (2) the impregnation of a catalyst precursor, and (3) the reduction of the catalyst. In this type of process, the sol–gel process is used to prepare the catalyst support layer with a high specific surface area; the catalyst precursor is usually a salt solution of the desired catalyst, and the catalyst reduction process is the reduction of the adsorbed precursor ions to metal monomers using a reducing agent [42]. Yue et al. [43] prepared a Pt/γ-Al_2_O_3_ catalyst layer in microreactors for catalytic methane combustion. In this case, the catalyst support precursor was first prepared by mixing γ-Al_2_O_3_ powers, binder, and acetic acid. Then, the obtained precursor was deposited on the walls of the microreactor using a syringe pump. After dehydration (120 °C for 8 h) and calcination (600 °C for 2 h), the support layer was prepared in the microreactor. Subsequently, incipient wetness impregnation was used to absorb the catalyst ions in a Pt(NH_3_)_4_(NO_3_)_2_ solution, and a subsequent calcination process was employed to reduce the catalyst ions. The obtained results demonstrated the superiority of the adhesion strength and catalytic activity for methane conversion. Meanwhile, it should be pointed out that the increase in the specific surface area of the support layer can play a positive role in improving catalyst dispersion and increasing the utilization efficiency of precursors. Hence, porogenic agents and structure-directing agents are usually employed in the preparation of support layers to form microporous, mesoporous, macroporous, or mixed–pore structures on the inner surface of microreactors [40,44,45]. By using F127 and P123 as the structure–directing agents and tetraethyl silicate and tetrabutyl titanate as the support precursors, Kataoka et al. [40,44] prepared a mesoporous silica and mesoporous titanium dioxide support layer with 2D and 3D structures in borosilicate glass capillaries with an inner diameter of 200 μm, respectively (see Figure 2c). The surface morphology showed that the obtained support layer allowed highly ordered structures with pores open to the surface. Benefitting from these specific structures that inherently provided a plentiful and ordered pore structure as well as abundant adsorption sites for the nanocatalysts, when platinum nanocatalysts were deposited on these structured catalyst support layers, excellent catalytic activity and selectivity as well as stability were obtained [46]. Apart from using a structure–directing agent to construct the pore structure, the template method was used by Guan et al. [47,48] to prepare a highly ordered macroporous structure of γ-Al_2_O_3_ support layer. Through introducing a γ-Al_2_O_3_ precursor sol containing polymethyl methacrylate particles (template), γ-Al_2_O_3_ particles, distilled water, and PVA, after calcination was used to remove the template and PVA, a γ-Al_2_O_3_ support with an inverse opal structure was obtained (see Figure 3). When palladium and platinum nanocatalysts were loaded on this support, an obvious intensification in methane conversion could be reached.

Although the catalytic activity and stability prepared by the above–mentioned sol–gel methods are satisfactory, it should be pointed out that to enhance the interaction between the catalyst and support, and between the support and reactor substrate, the calcination process is typically essential. To satisfy this requirement, considering that the heterogeneous reaction is diverse and the material requirements of the reactor are also different, the selection of the substrate materials for microreactor fabrication then faces severe challenges. This section is divided into subheadings. It provides a concise and precise description of the experimental results and their interpretation, as well as the experimental conclusions that can be drawn.

## 3. Bio–Inspired Electroless Deposition Method

**Figure 4:**
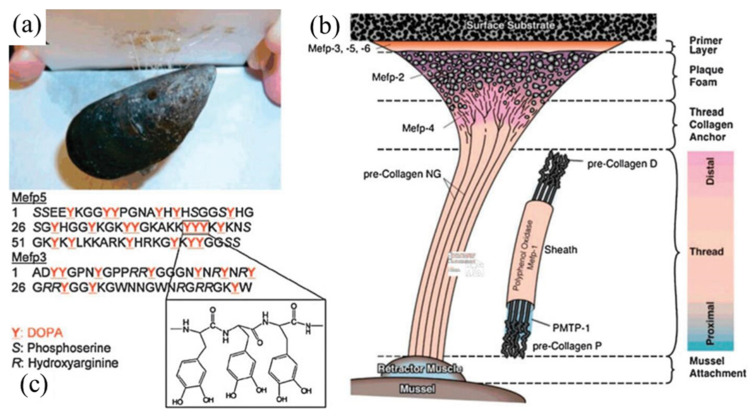
(**a**) Local adhesive–related proteins of blue mussels binding to Teflon, (**b**) adhesive−related proteins in the byssus of mussel, (**c**) the amino acid sequences of Mefp−3 and Mefp−5, adapted from [49], Copyright 2011, with permission from RSC.

Under a humid environment, the anchor foot silk thread of marine mussels will secrete a kind of adhesive protein, which means that marine mussels can firmly adhere to a reef surface [49], as shown in Figure 4A. Inspired by this, Lee et al. [50] investigated the adhesion properties of 3,4–dihydroxy–L–phenylalanine and lysine, which are important components in viscosity proteins, and found that these substances can adhere to almost any surface, including organic/inorganic material surfaces, metal–nonmetals, solid oxides, and polymers [51]. As an important derivative of 3,4–dihydroxy–L–phenylalanine, dopamine inherently allows both amino and phenolic hydroxyl groups, meaning it can produce similar properties to adhesive proteins. Thus, mussel–inspired adhesives and coatings have attracted extensive attention from researchers around the world [52,53,54].

**Figure 5:**
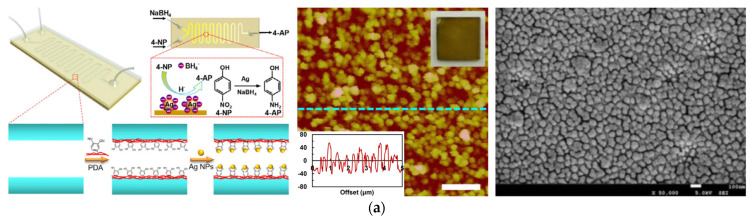

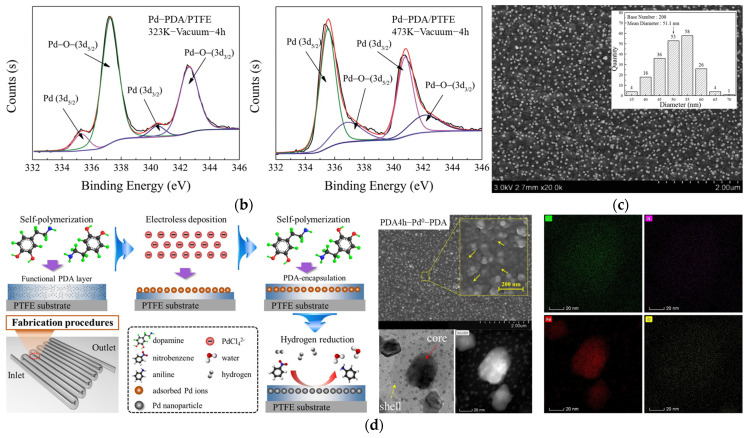
(**a**) Schematic illustration of the deposition of Ag nanoparticles in microreactor through bio–inspired electroless deposition and the corresponding AFM and SEM images of the prepared catalyst layer, adapted from [58], Copyright 2016, with permission from Elsevier. (**b**) XPS results of Pd−PDA/PTFE catalyst layer without and with hydrogen reduction, adapted from [56], Copyright 2018, with permission from RSC. (**c**) SEM images and Pd nanocatalyst size distribution of Pd−PDA/PTFE catalyst layer with hydrogen reduction, adapted from [13], Copyright 2016, with permission from Elsevier. (**d**) Schematic description of the fabrication of the core–shell structured catalyst layer and the corresponding surface morphology and element distribution, adapted from [60], Copyright 2019, with permission from Elsevier.

Through self–polymerization under alkaline aerobic conditions, dopamine can form a polydopamine (PDA) layer on almost all substrate materials and irregular surfaces [55,56,57]. Thanks to its rich amino and phenolic hydroxyl functional groups, PDA coatings can firmly bond to a substrate surface through covalent bonds or other intermolecular forces. In addition, these amino and phenolic hydroxyl groups also allow polyamine to show a certain reduction ability [58]. Ascribing to these merits, the PDA layer then can show a variety of functional applications, such as surface treatment, water filtration, biological analysis, electroless deposition, etc. Among them, electroless deposition is a simple, rapid, and environmentally friendly method for the preparation of a catalytic layer based on the adsorption and reduction of metal ions by functional groups on the surface of the polyamine layer. Zhang et al. [58] successfully deposited a Ag nanocatalyst on the inner wall of a glass microchannel by modifying the inner surface of the glass microchannel with PDA, as shown in Figure 5a. The prepared catalyst layer showed a high catalytic activity for the catalytic reduction of 4–nitrophenol (4–NP). Ryoo et al. [59] prepared peptide nanowires on the inner wall of a homemade polysilazane microchannel reactor. By modifying the peptide nanowires with PDA, well–dispersed palladium nanocatalysts could then be deposited on the surface of peptide nanowires and presented an excellent catalytic activity for various hydrogenation reactions. In addition to depositing a single component catalyst, Ye et al. [57] synthesized Pt–Au dendrimer–like nanoparticles on a PDA–functionalized surface and a stable conversion efficiency of around 100% over six successive reduction cycles of 4–NP reduction was obtained.

**Figure 6:**
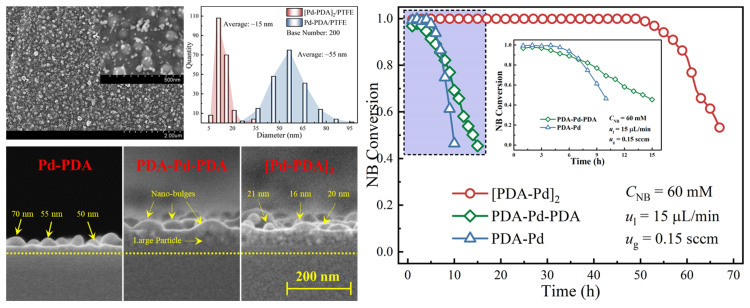
Surface morphology and particle size distribution of nano–bulge structured catalyst layer and the corresponding catalysis performance and durability for NB hydrogenation, adapted from [62], Copyright 2018, with permission from Elsevier.

However, it is worth noting that the reduction of the adsorbed catalyst precursor ions mainly relies on the reducing groups in the PDA layers. Due to the limited reduction ability of these reducing groups in PDA, there still exists a large number of residual metal ions on the surface (see Figure 5b), which significantly decreases the utilization efficiency of the absorbed metal ions [56,61]. To solve this problem, Feng et al. [13] developed a serpentine microreactor with a Pd nanocatalyst deposited on the inner surface of a PDA–coated microchannel. The conventional hydrogen reduction process was introduced into the catalyst layer preparation procedure to decrease the residual Pd ions. The obtained results showed that most of the palladium ions were successfully reduced, and a mean particle size of about 50 nm was obtained (see Figure 5c). Although the prepared catalyst layer in the microreactor exhibited a rather high nitrobenzene (NB) conversion of over 97% and an excellent aniline (AN) selectivity of nearly 100%, the large size of the palladium particles formed during the reduction process still inhibited the utilization efficiency of the deposited catalyst. Hence, in the same research group, as shown in Figure 5d, a core–shell structured palladium nanocatalyst layer was proposed in a microreactor [60]. Via encapsulating the active palladium species absorbed on the PDA layer with another PDA shell, the constructed barriers between the adjacent palladium particles could then not only restrain the migration and aggregation during the reduction and reaction processes but also played a positive role in protecting the palladium cores from being leached. These merits then made the formation of large particle sizes be restrained and a 120% improvement in terms of durability could be obtained. In addition, another strategy for decreasing the particle size is to construct nanostructures on a surface to enlarge the loading area and accessible sites for precursor ion deposition. Zhu et al. [62] proposed a multilayered palladium nanocatalyst with a nano–bulge structure inside a microreactor. Benefitting from the large particle sizes at the bottom, the unique bulged nanostructure could play positive roles in providing more accessible sites for precursor adsorption and extending the available area to prevent catalyst aggregation. As a result, a 35.4% higher catalyst loading and a four–fold smaller catalyst size were simultaneously realized. These advantages then significantly intensified the catalytic activity and allowed an order of magnitude longer durability (see Figure 6). In addition to building a nanostructure, Chen et al. [54] first deposited metal–organic frameworks (ZIF–8) in polyethersulfone membrane pores that were modified by PDA and then immobilized a Ag nanocatalyst on the membrane via ion exchange to construct a membrane microreactor. The introduction of ZIF–8 significantly decreased the size of the Ag nanocatalyst, and the apparent reaction rate constant reached a remarkable intensification by two orders of magnitude.

The above research shows that the preparation of a catalytic layer by bio–inspired electroless deposition has become one of the potential and competitive catalytic layer preparation technologies in microreactors. However, it should be pointed out that compared with the traditional sol–gel method or impregnation–reduction method, the particle size of catalysts is still too large to meet the economic requirements of industries. Meanwhile, considering that the catalytic performance is not only determined by the particle size, the crystalline structure, coordination structure, surface structure, mechanical strength, etc., can also play significant roles in the catalytic performance. More attention should also be paid to these properties. Therefore, catalyst layers prepared by this method still have various problems.

## 4. Layer–by–Layer Self–Assembly Method

**Figure 7:**
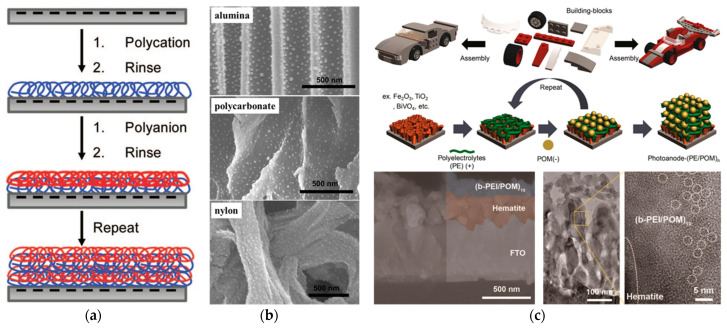
(**a**) Schematic diagram of constructing the multilayered film using LBL, adapted from [64], Copyright 2008, with permission from ACS. (**b**) Cross−sectional images of polyaion/PAH/Ag nanoparticle films on different materials, adapted from [66], Copyright 2008, with permission from ACS. (**c**) Layer−by−layer modified photoanode for photoelectrochemical water splitting, adapted from [70], Copyright 2008, with permission from ACS.

Layer−by−layer self-assembly (LBL) is a method of making multilayer films by alternately depositing charged polymer polyelectrolytes, which is driven by electrostatic interactions between molecules or ions with opposite charges [63,64]. The schematic diagram of constructing a multilayered film is shown in Figure 7a. Firstly, the surface modification method is used to make the surface of the reactor substrate charged. Then, employing a water–soluble polyelectrolyte with an opposite charge to the substrate surface, a polyelectrolyte layer forms in the substrate surface through electrostatic adsorption. Finally, by alternately depositing the polyelectrolytes with opposite charge properties, multilayered polyelectrolyte films then can be obtained on the surface. Since the polyelectrolyte layer inherently allows positive or negative surface charge properties, based on the charge property of the catalyst precursor ions, the ions can be immobilized on the polyelectrolyte surface through electrostatic adsorption. Under this circumstance, a wall–coated catalyst then can be obtained after the reduction step [65]. Ascribing to the advantages of being flexible, simple, controllable, environmentally friendly, shape and material independent, etc., these superiorities of the LBL method have then attracted extensive attention of researchers, and various nanocatalysts including inorganic nanoparticles (e.g., Pd, Au, Ag), carbon nanotubes, dendrimers, polypeptides, etc., have been deposited in microreactors and applied for various heterogeneous catalytic reactions [66,67,68,69].

Typical research was proposed by Dotzauer et al. [71], where gold nanoparticles were deposited on a catalytic membrane modified with PAA/PAH polyelectrolytes (see Figure 7b). The SEM results showed that a high density of wel–separated nanocatalysts was obtained in the membrane pores, which presented excellent catalytic activity for nitroaromatic reduction. Jeon et al. [70] applied this LBL method to the preparation of photoelectrodes for photocatalytic water oxidation. By depositing a thin film of various cationic polyelectrolytes and anionic polyoxometalate catalysts on the surface of photoelectrode materials (see Figure 7c), the stability and photocatalytic properties obtained a remarkable improvement. De León et al. [72] built a polyelectrolyte membranes (PEMs) composite film incorporated with alkaline phosphates to obtain a higher catalytic activity of enzymatic catalytic microreactors; this opened the route for enzymatic catalysis applications in microreactors. PEMs serving as support to fix metal nanocatalysts in a microchannel were also used by Kanungo et al. [73], where, they first deposited a silica precoat layer by the sol–gel method, and then the LBL assembly method was implemented for obtaining PEMs by alternatively flushing solutions of polyelectrolytes through these microchannels using a syringe pump. Finally, a charged surface that was ideal for metal ion adsorption and metal nanoparticle anchoring was formed. Liu et al. [74] designed a plate–type catalytic membrane microreactor and prepared palladium nanocatalysts on a PEM–functionalized polydimethylsiloxane membrane. The obtained catalyst layer also showed excellent performance for NB conversion. Apart from these common heterogeneous catalytic reactions, Khositanon et al. [75] also immobilized a photocatalyst (TiO_2_) onto the wall of a perfluoroalkoxy alkane tube by initially depositing a polydopamine and polyethyleneimine layer. Through alternate LBL deposition, a high photocatalyst loading was realized to enhance photocatalytic methylene blue degradation.

**Figure 8:**
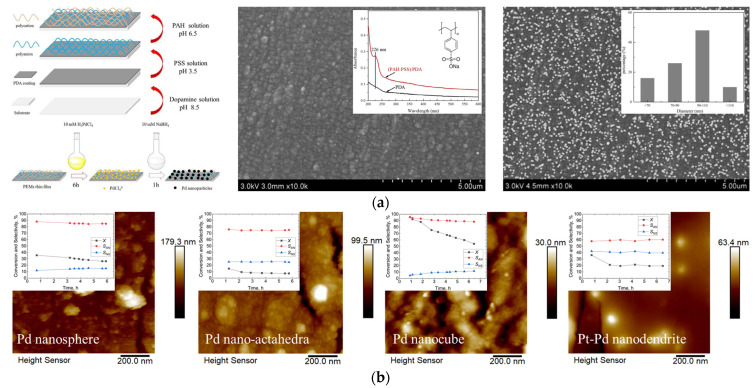
(**a**) Schematic diagram of constructing a multilayered film on inert materials and the surface morphology before and after catalyst deposition, adapted from [77], Copyright 2018, with permission from Elsevier. (**b**) AFM results of depositing ex situ synthesized Pd nanocatalyst with different nanostructures on PEMs and corresponding catalytic performance, adapted from [79], Copyright 2021, with permission from Elsevier.

In addition, considering that a variety of heterogeneous catalytic reactions that inherently present highly corrosive, inert materials such as PTFE have been widely used as the material of the reactor body or lining in these reactions [56,76], it should be pointed out that it is typically hard to create a charged surface using these materials, and thus the above–mentioned bio–inspired surface modification method was introduced into the LBL method to further expand its application scope. For example, using a PTFE capillary tube with an inner diameter of 600 μm as the microreactor substrate, Liu et al. [77] first prepared a PDA layer on the inner surface of the PTFE capillary tube. Since a PDA layer can present a positive potential at low pH values [19], an anionic solution containing 0.02 M PSS and 0.5 M NaCl (pH adjusted to 3.5) was then introduced into the capillary tube modified with the PDA layer to deposit the PSS layer. Then, a negatively charged surface was obtained in the microreactor. Following the modification of the cationic polyelectrolyte PAH, a thin (PAH–PSS)/PDA film was fabricated in the microreactor, whereas regarding the catalyst deposition, an aqueous H_2_PdCl_4_ solution was used as the precursor and was introduced in the PTFE capillary modified with (PAH–PSS)/PDA film. Since the PdCl_4_^2-^ ions in the solution could bind to the amino group [−NH_3_^+^], the palladium nanocatalyst with a high dispersion on the (PAH–PSS)/PDA film of the microreactor could then be obtained with the help of NaBH_4_ reduction (see Figure 8a). Both a high conversion efficiency and selectivity as well as stability were obtained for the catalytic hydrogenation of NB to AN [78]. Venezia et al. [79] also deposited a PDA coating on the inner surface of a tubular Teflon AF–2400 membrane followed by the sequential grafting of PEMs and the ex situ synthesis of Pd nanoparticles (see Figure 8b). Such nanostructured metallic particles can be immobilized on a PEM support coating inside microreactors and show a desirable catalytic performance for heterogeneous reactions.

It can be seen from the above summary that LBL inherently presents the merits of being flexible, efficient, convenient, etc. Since this method is not limited by the reactor material, shape, and size, it is particularly suitable for catalyst layer preparation in small devices, such as microreactors. However, it should also be pointed out that, similar to the bio–inspired electroless deposition method, the important properties of the catalyst including particle size, crystalline structure, etc., are still not controllable. Hence, more related works are still urgently required to solve these issues.

## 5. Summary and Outlook

As a promising technology, microreactors show unique advantages for heterogeneous catalytic reactions and have attracted tremendous attention worldwide. This review provided a general description of the physicochemical processes of heterogeneous catalytic reactions in microreactors and systematically summarized the recent advances in catalyst layer preparation in microreactors. Particular attention was focused on the most common sol–gel method and its development. In addition, some new strategies proposed recently, including bio–inspired electroless deposition and LBL, were also comprehensively discussed. From the reported results of the literature, the proposed catalyst layer preparation methods can provide satisfactory catalytic activity and stability for heterogeneous catalytic reactions. However, compared with commercial catalysts, these methods are still far from industrial deployment. A typical challenge is catalyst utilization, since single–atom catalysis has made great progress [80,81,82]. In fact, this issue is more serious in the bio–inspired electroless deposition method and LBL method, because the catalyst particle size prepared by these two methods is in the order of 10^1^~10^2^ nanometers. Hence, more in–depth research focusing on the structural design of the support layer and surface modification should be carried out to provide more surface area and accessible sites (or functional groups) for optimizing the catalyst utilization efficiency. In addition, the performance of a considerable part of heterogeneous catalytic reactions also depends on the crystalline structure of the catalyst; however little attention has been paid to the crystal surface control of catalysts deposited in microreactors. As a result, we anticipate that this methodology research could play a positive role in further expanding the application scope of microreactors and promoting their industrialization.

In summary, various methods have been proposed to prepare highly efficient catalysts in continuous microreactors. However, there still exist some issues that need to be solved to promote their industrial application. Significant research breakthroughs in the design and control of both the particle size and crystalline structure of catalysts are still required for realizing efficient and stable heterogeneous catalysis in microreactors.

## Data Availability

Not applicable.
